# Portland Cement/*Acrocomia Aculeata* Endocarp Bricks: Thermal Insulation and Mechanical Properties

**DOI:** 10.3390/ma13092081

**Published:** 2020-05-01

**Authors:** Camila C. Calvani, Além-Mar B. Goncalves, Michael J. Silva, Samuel L. Oliveira, Bruno S. Marangoni, Diogo D. dos Reis, Cicero Cena

**Affiliations:** 1 Programa de Pós-Graduação em Ciência dos Materiais, Instituto de Física, UFMS—Universidade Federal do Mato Grosso do Sul, Campo Grande-MS 79070-900, Brazil; calvanicamila@gmail.com (C.C.C.); alem-mar.goncalves@ufms.br (A.-M.B.G.); samuel.oliveira@ufms.br (S.L.O.); bruno.marangoni@ufms.br (B.S.M.); diogo.reis@ufms.br (D.D.d.R.); 2 UNESP - Universidade Estadual Paulista “Júlio de Mesquita Filho”, Rosana-SP 19272-000, Brazil; michael.silva@unesp.br

**Keywords:** composite, *Acrocomia aculeata* (Jacq.) Lodd fruit, portland cement, mechanical properties, thermal insulation

## Abstract

In the last few decades, Portland/residue composites have been researched due to their technological and environmental advantages. In this study, residues of *Acrocomia aculeata* (Jacq.) Lodd endocarp (AE) were introduced in the Portland cement–soil (PC) matrix in different concentrations (0, 5, 10, 15, 20, and 50 wt%) to produce PC/AE bricks. The characterization of the microstructures of the bricks indicate agglomerates of AE particles with increased humidity in small regions distributed throughout the matrix. Mid-infrared and laser-induced breakdown spectroscopy, along with thermogravimetry, indicated that AE contained mainly lignin and cellulose, as well as inorganic chemical elements such as Mg and Si. X-ray studies revealed that AE did not affect the crystallographic properties of the Portland/AE bricks. The findings indicate that the use of AE improved the thermal insulation capability of the composites with a small impact on the compressive strength.

## 1. Introduction

Studies have been conducted involving the introduction of different residues in concrete and soil–cement bricks to produce composites with improved mechanical and physical properties [1,2,3] and manage residue from the environment [4]. Soil–cement brick is a component of masonry made of a homogeneous, compacted, and hardened mixture of soil, Portland cement, and water [5]. The insertion of materials composed of cellulose and lignin in Portland cement composite (PC) matrices is common. The addition to these materials, the cementitious matrix has high commercial importance, as they act as a filler and reduce the amount of cement needed in the matrix. The procedure also has a beneficial effect on the environmental and economic matters, as it reuses waste material, adding value to the residue.

Coconut and peanut shells introduced in PC matrices have allowed producing composites with interesting durability properties but low resistance [4]. The insertion of rice husk ashes yielded a decrease in compressive strength, followed by an increase in the durability of the concrete and reduction of porosity [3]. The application of sugarcane bagasse influenced the hydration reactions of the cement, behaving like a non-plastic material with moderate pozzolanic activity, and their particles contributed to improving the packaging characteristics of soil-cement mixtures [6]. 

In general, the studies have shown wood/cement composites with low density, lightweight, certain deformation response, improved acoustic properties, and better thermal insulation capacity. However, this comes at the cost of the reduced mechanical strength of the composite [7,8].

**Figure 1:**
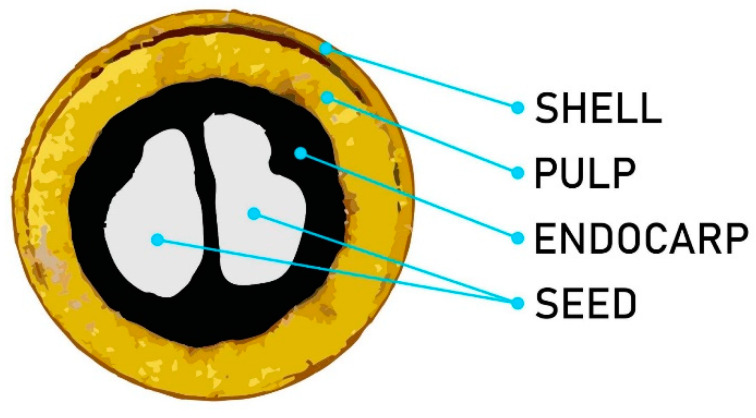
Illustration of *Acrocomia aculeata* (Jacq.) Lodd fruit and its parts.

The *Acrocomia aculeata* (Jacq.) Lodd fruit, also known as bocaiúva, coco-babassu, coco-de-espinho, macaíba, macaibeira, and macaúba, comes from a palm found in many regions of the Americas. These palms are distributed mainly in the Cerrado and Pantanal biomes. In Brazil, the *Acrocomia aculeata* endocarp (AE) fruit is the leading commercial product of the palm. It has been employed in biodiesel and animal feed, among other food products. The fruit consists of pulp (mesocarp), fibrous material with a sweet flavor, endocarp firmly attached to the pulp, and nut (Figure 1) [9].

The endocarp is composed of the sclerenchyma cells that have cell walls made of lignin, cellulose, and hemicellulose. The existence of lignin becomes the cell wall impermeable, forming a barrier of high mechanical resistance to protect the *Acrocomia aculeata* seed until the bud phase [9]. The endocarp has a high concentration of lignin; thus, it presents a heat absorption capacity higher than one exhibited by wood [10]. Besides, in the regions where the fruit is more common, as an energy source for domestic consumption and, on an industrial scale, for coal production. 

In this study, bricks made of Portland cement and *Acrocomia aculeata* endocarp were produced by manual compaction using a wood mold. The effect of the substitution of Portland cement by endocarp on the thermal conduction and mechanical strength of the bricks was studied. Additionally, microstructural properties of the samples were investigated to a better understanding of the thermal insulation and mechanical strength results.

## 2. Materials and Methods 

### 2.1. Raw Materials and Sample Preparation

AE were obtained in Campo Grande, Mato Grosso do Sul, Brazil, and washed in running water to remove impurities. The AE was heated in a furnace at 250 °C for 10 h with a heating rate of 5 °C min^−1^. After cooling, the material was milled for three hours to achieve the granulometry recommended by either the standard NBR 7211 [11] or particle size below 0.075 mm. 

The commercial soil of Campo Grande-MS with 1.38 Fineness Modulus and the commercial Portland cement (PC) were used to produce the bricks. CPII-Z 32 cement (Portland cement with pozzolana) was employed, which exhibits a compressive strength of 32 MPa at 32 days of cure. Tap water, free of organic matter, was employed, constituting 10 to 15 vol% of the total solids of the mixture [12,13].

The composites were produced with the standard dimensions (200 × 100 × 50 mm^3^) using soil, PC, AE, and water by manual compaction in a wood mold [5]. Portland cement was replaced by AE to obtain composites with 0, 5, 10, 15, 20, and 50 wt% of AE in the total mass. Although the sample with 50 wt% AE did not present mechanical resistance, it was useful for the analyses of the properties of the entire set of PC/AE composites. Six bricks of each composition were prepared. They were placed on a rigid horizontal surface, free of vibrations, for 24 h after molding. Then, the bricks were removed from the molds and immersed in water for curing. All tests performed followed the technical standard NBR 12024/2012 with the cure of 7, 14, and 28 d. Two specimens were taken in these days to test the effect of AE on the compressive strength of the bricks [14].

### 2.2. Sample Characterization

Surface and cross-section analysis of the PC/AE composites was done by using optical microscopy (Bioptika model B100 Series, Phox; Colombo, Brazil). X-ray diffraction of the composites was carried out using a Shimadzu diffractometer (Co radiation, model 6100, Shimadzu; Kyoto, Japan) with a 2θ interval from 10° to 80°, and a rate of 0,02°∙min^−1^.

Attenuated total reflectance Fourier transform infrared spectroscopy (ATR-FTIR) was used to evaluate the presence of AE in the bricks. The measurements were performed from 4000 to 600 cm^−1^ with 4 cm^−^¹ resolution and 16 scans. Thermogravimetric (TG) measurements were also performed by a TGA Q50 (TA Instruments, New Castle, USA) from 40 to 800 °C (10 °C∙min^−1^) in a nitrogen atmosphere (50 mL∙min^−1^). 

The laser-induced breakdown spectroscopy (LIBS) [15] of the AE sample was performed using a Nd:YAG laser with 200 mJ per pulse and 10 ns temporal width. A spectrometer (Stellarnet, Florida, USA) operating from 190 to 300 nm with a 0.2 nm optical resolution was used. The 500-ns delay time between the plasma formation and spectrum acquisition was adopted. The spectra were acquired by averaging the spectra from two consecutive shots after a previous shot for surface cleaning. Fifteen spectra were obtained for each sample (inner and surface) and underwent a process to exclude outliers and were averaged [16,17].

Mechanical characterization was performed according to the standard NBR 8492 [18] utilizing a Forney F-40 DR mechanical (Forney; Zelienople, USA) press with a uniform load rate of 500 N/s. This test was performed by using two samples for each curing period. Each sample was cut perpendicularly to the length, and then the parts were placed on each other and connected by using cementitious paste. Finally, the samples were pressed until its rupture, and the value of compressive strength was calculated, taking into account the average value of both samples.

The relative thermal conductivity was estimated using the direct thermal-comparator method [19,20]. This approach is appropriate for analyzing cementitious materials due to its dimensions, in which a metal wire surrounded by a thermal insulation material connects the heat reservoir to the sample. The temperature difference between the heat reservoir and the wire tip in contact with the sample was determined. The relative thermal conductivity was calculated by comparing the temperature difference in the composite with the one observed in the PC brick. Five bricks of each composition were measured at 28 d of curing. 

## 3. Results and Discussion

**Figure 2:**
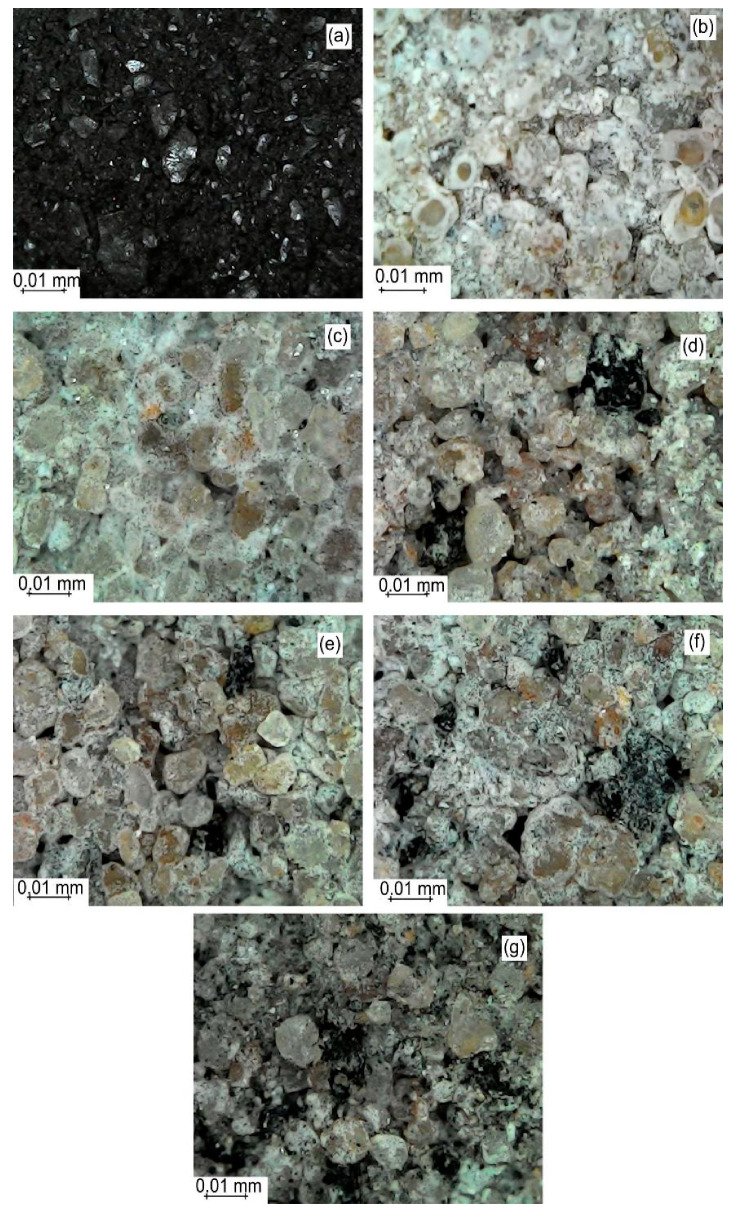
Surface images of the (**a**) thermal treated *A. aculeata* endocarp (AE) and cross-section of Portland cement composite (PC)/AE composites with (**b**) 0, (**c**) 5, (**d**) 10, (**e**) 15, (**f**) 20, and (**g**) 50 wt% AE.

Figure 2 shows the typical morphology of the thermal treated AE and PC/AE composites. The AE particles exhibited a dark appearance as a result of the treatment at 250 °C. The surface of PC/AE bricks with 0, 5, 10, 15, 20, and 50 wt% AE revealed PC-soil clusters with a diameter of about 10 µm. The soil particles in the sample without AE were surrounded only by Portland cement (Figure 2b). In its turn, the soil particles were less surrounded by PC with the AE addition in the matrix. Besides, the regions occupied by AE particles were still moist even long after the bricks were removed from water due to the AE ability to absorb moisture [9]. 

**Figure 3:**
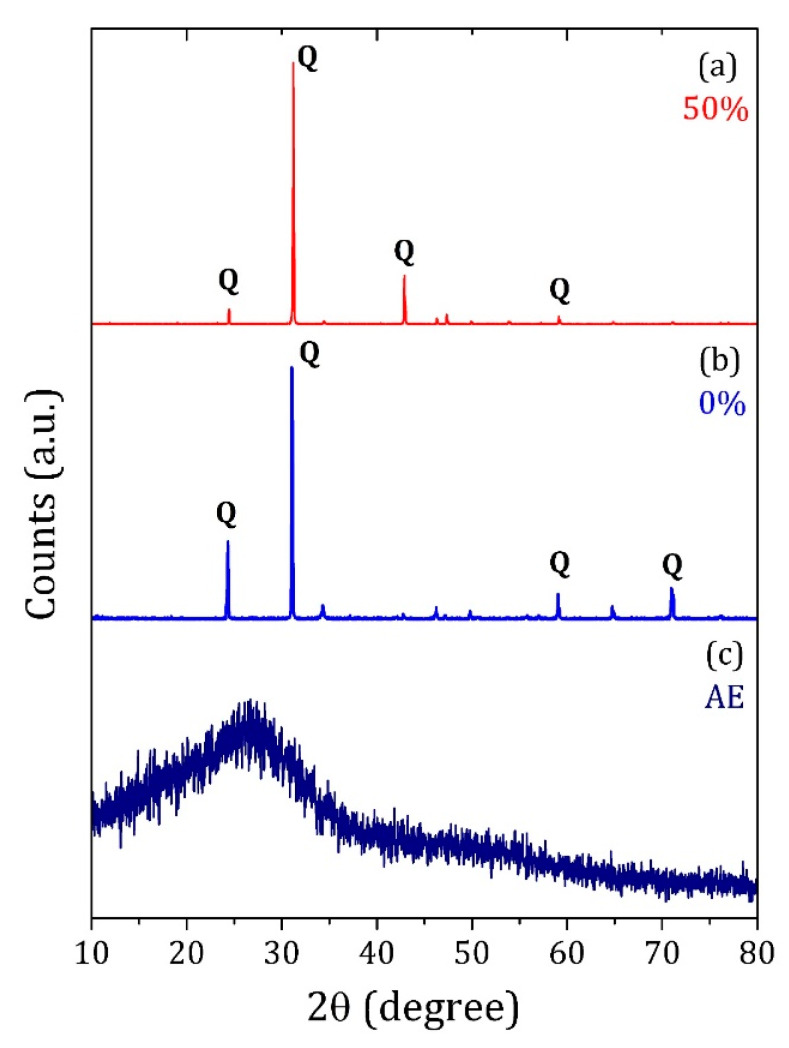
X-ray diffraction pattern of the (**a**) PC/AE composite with 50 wt% AE, (**b**) PC sample, and (**c**) AE particles after thermal treatment at 250 °C.

The X-ray diffraction pattern of the AE and PC/AE composites are exhibited in Figure 3. The AE powder showed no crystalline phases after thermal treatment, with a characteristic large amorphous halo at approximately 27°. The PC and PC/AE bricks with 50 wt% AE presented a crystallographic phase related to SiO_2_ (Quartz) with small differences in the relative peak intensities [21]. Increasing the AE particles in the PC/AE composite did not substantially affect the crystallographic characteristics of the matrix.

**Figure 4:**
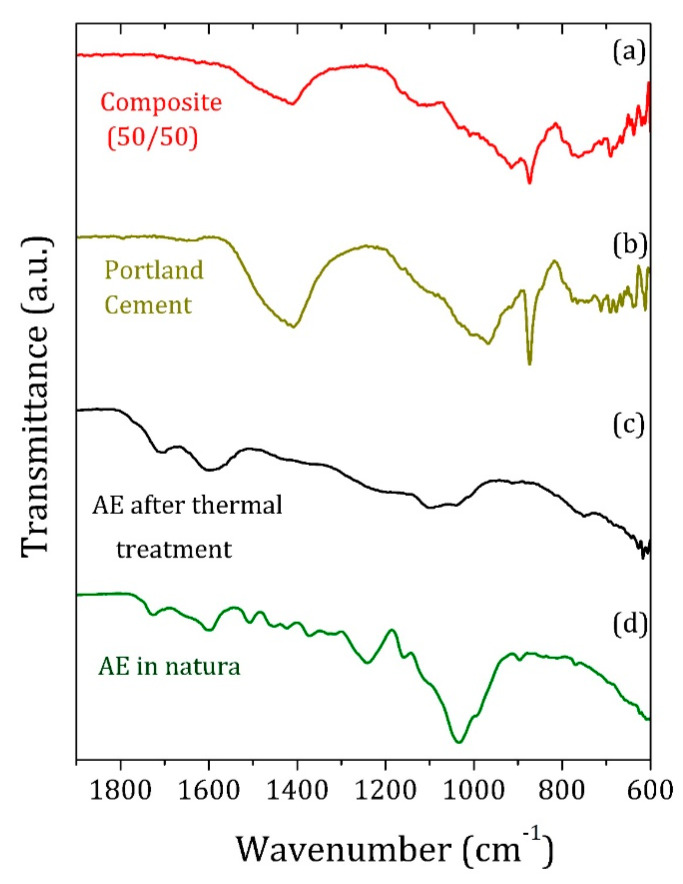
Infrared spectra of the (**a**) PC/AE composite with 50 wt% AE and (**b**) PC sample, both after 28 d of cure, (**c**) AE particles after thermal treatment at 250 °C, and (**d**) AE in natura.

The FTIR measurements of unheated AE (in natura) revealed major absorption bands associated with lignin and cellulose compounds (Figure 4). After thermal treatment, a substantial decrease in the band’s intensity is observed. Bands even disappeared possibly because of the thermal degradation of organic compounds such as the lignin and cellulose molecular chains.

Table 1 shows the assignments for each mid-infrared absorption band. The infrared band at around 1410 cm^−1^ is associated with the asymmetric C–O elongation of the CO_3_^−2^ group in calcium carbonate groups [22], while C–O and Si–O vibrations between 915 and 775 cm^−1^ are related to the soil minerals [22,23,24]. Even with a large amount of AE in the matrix, both lignin and cellulose were not detected clearly in the PC/AE composites via infrared spectra; only a few differences in the 1000–1200 cm^−1^ range could be associated with organic material.

**Figure 5:**
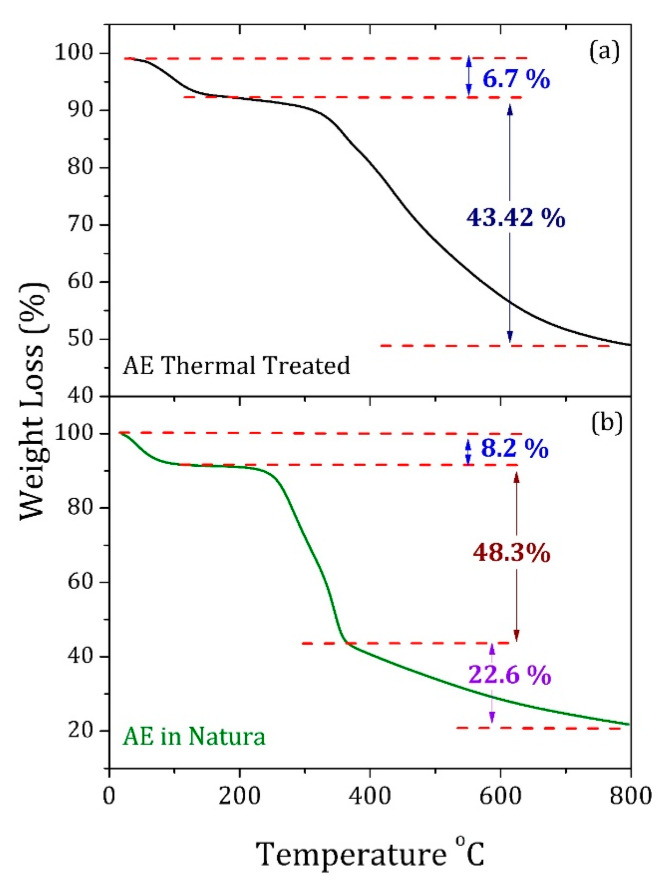
Thermogravimetric (TG) curve of the (**a**) thermal treated and (**b**) untreated AE.

A thermogravimetric (TG) study was performed to investigate the thermal degradation of the AE compounds. Figure 5 shows the degradation curve as a function of the temperature for the samples thermally treated at 250 °C for 10 h as well as the untreated one. The TG curve of the untreated AE sample exhibits three main steps. The first step starts at room temperature and ends at approximately 210 °C, with a weight loss of 8.2% related to the release of free and structural water [25]. At the second step, lignin and cellulose chains degraded in the 210–370 °C range with a weight loss of 48.3%. The third step, corresponding to the interval between 370 to 800 °C with a total loss of 22.6 wt%, could be associated to the cellulose degradation, remaining 20.9% of the total mass [26,27].

The TG curve of the heated AE at 250 °C/10 h shows only two main degradation steps, the first one linked to the release of structural water with the weight loss of 6.8% between 50 and 210 °C. The second step, with the weight loss of 43.2% in the 210–800 °C range, was a result of the degradation of lignin and cellulose chains. In the end, about 49.8% of the total mass remained. Therefore, these results imply that organic compounds and inorganic elements, which do not degrade after 800 °C, still were present in the heated AE. 

**Figure 6:**
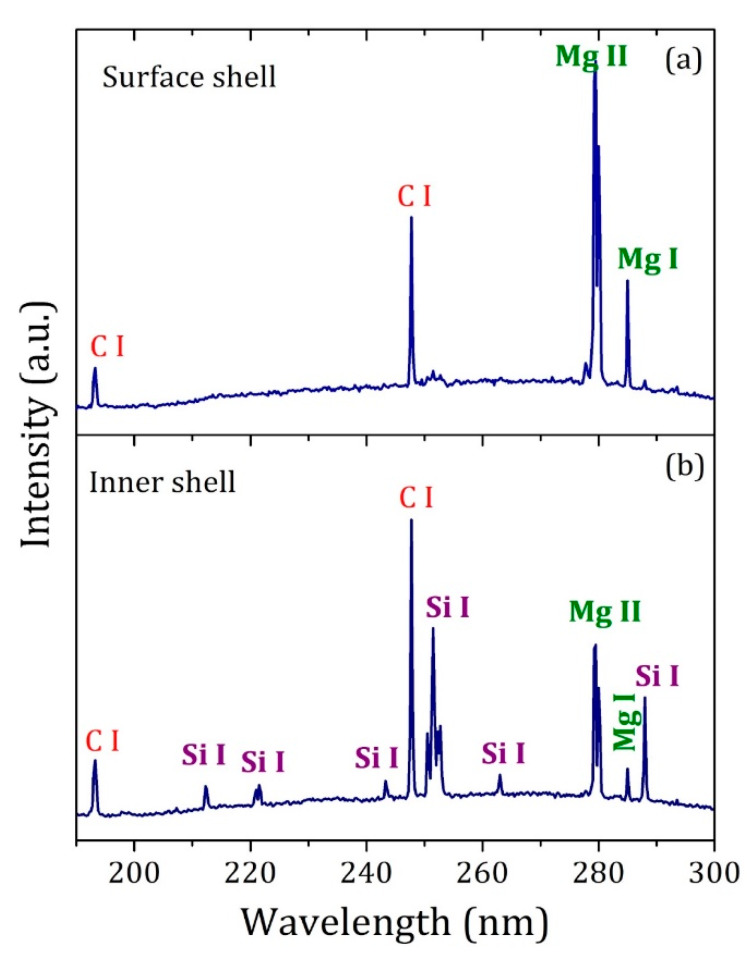
Laser-induced breakdown spectroscopy (LIBS) spectra obtained on the (**a**) surface and (**b**) inside the AE in natura.

The LIBS analysis confirmed the hypothesis of the presence of carbon and inorganic elements in the AE sample in natura (Figure 6). The elements C and Mg were identified as the main constituents of the surface of the AE (surface shell), while the Si element was detected in the inner shell along with C and Mg.

**Figure 7:**
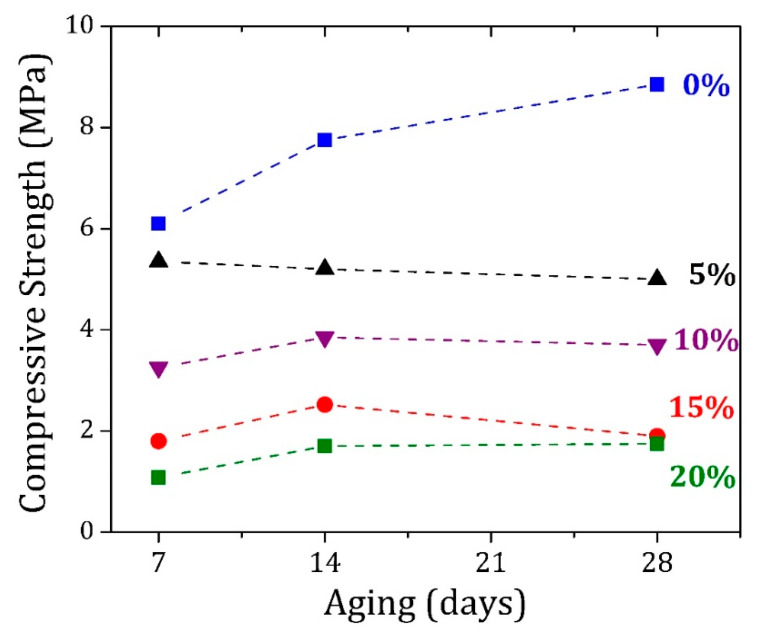
Compressive strength of the PC and PC/AE bricks for different ages.

The compressive strength values of the PC and PC/AE bricks in different cure times are highlighted in Table 2 and displayed in Figure 7. The mechanical resistance of the bricks decreased as a function of the amount of AE in the composite matrix. Once the Portland cement is mainly responsible for the mechanical strength of the bricks acting as a binder, the substitution of PC by AE promotes a minor percolation of the PC in the matrix, modifying the mechanical properties of the brick. The usual behavior of compressive strength for pure PC brick is shown in Figure 7. The value of compressive strength was enhanced by about 45% from 7 to 28 d of curing due to the calcium-silicate phase formation during this period. This value should grow four times longer than 28 d. The rising tendency with curing time was observed only for the sample with 20 wt% AE, in which the compressive strength increased around 62% in the same period. On the other hand, the sample with 5 wt% AE showed the opposite behavior, with compressive strength decreasing 6% between 7 and 28 d of curing. Although the samples with 10 and 15 wt% AE exhibited an improvement of 14 and 5%, respectively, in the compressive strength during the entire period, a decrease of 4 and 25% was observed from 14 to 28 d of curing, respectively. An experimental deviation due to the standard procedure recommended by NBR 8492 cannot be ruled out because it is based on an average value from two samples, which could be affected, e.g., by the molding before insertion of the sample in the mechanical press or intrinsic differences among the samples related to the manufacturing.

According to NBR 8491 standard [5], the average compressive strength of the brick must not be lower than 2.0 MPa (20 kgf∙cm^−2^), with no individual value lower than 1.7 MPa (17 kgf∙cm^−2^), for a minimum of 7 days of curing. PC/AE composites with 10 wt% AE or less met the requirements, while the other samples were affected by the reduced PC concentration, impacting their mechanical resistance [18].

**Figure 8:**
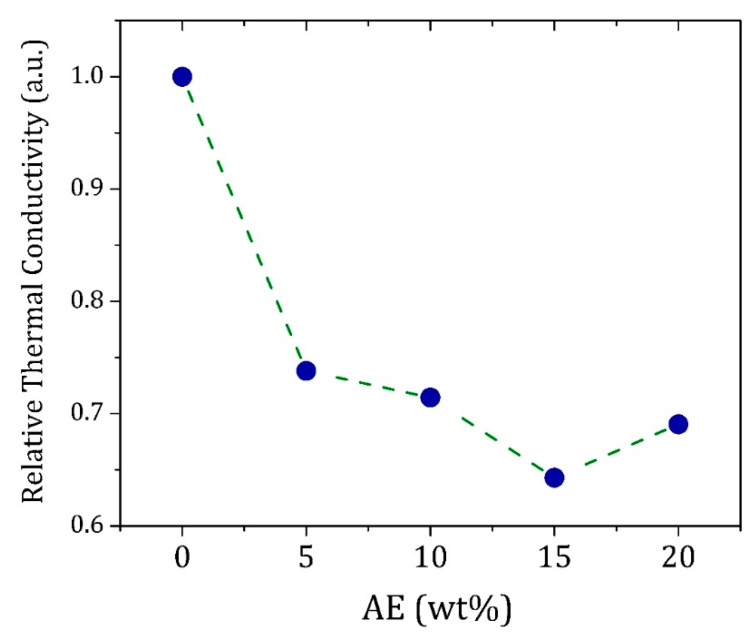
Relative thermal conductivity of the PC and PC/AE composites with 28 d of curing.

The thermal conductivity of the composites was determined, considering the PC brick as reference (Figure 8). The composite with 5 wt% AE presented a thermal conductivity 25% lower than one verified in the PC material, but the higher reduction (37%) was confirmed for the composite with 15 wt% AE. The significant changes in the thermal conductivity should be associated with the AE clusters dispersed in the matrix, which act as heat absorption sites due to their intrinsic high heat capacity.

## 4. Conclusions

Composites made of Portland cement-soil and *Acrocomia aculeata* endocarp were produced, and their mechanical and thermal properties were analyzed. The use of 5 wt% AE in the composite was enough to provide a material with a thermal conductivity of 25% lower than one verified in the PC bricks. Above 5 wt% AE, it was observed no improvement of thermal insulation and a significant decrease of compressive strength. PC/AE composites with 10 wt% AE or less fulfilled the minimum requirements for civil construction with improved thermal insulation capacity. Therefore, they could be used in civil construction, especially for thermal insulation purposes.

## Figures and Tables

**Table 1:** Assignments of the FTIR bands of the samples [22,23,24].

Chemical Compound	Assignments	Wavenumber (cm^−1^)
Lignin	C=O axial deformation	1724
		1708
	C=C and C=O stretching	1600
	C–H bending	893
		752
Hemi-Cellulose and Cellulose	C=C and C=O stretching	1509
	C-H scissoring	1371
	C–O axial deformation	1238
		1212
	C–O–C asymmetric stretch	1160
	C–O stretching	1100
		1034
	C–O asymmetric stretch	1410
Portland cement composite	C–O stretching	1100
	C–S–H asymmetric stretch	967
	C–O asymmetric stretch in Calcium Carbonate; Si–O stretching	915–775

**Table 2:** Compressive strength of the PC and PC/AE composites at 7, 14, and 28 d of curing.

AE in PC/AE Samples (wt%)	Sample Age/Compressive Strength (MPa)		
	7 Days	14 Days	28 Days
0	6.10	7.75	8.85
5	5.35	5.20	5.00
10	3.25	3.85	3.70
15	1.80	2.52	1.90
20	1.08	1.70	1.75
